# *Cia5d *regulates a new fibroblast-like synoviocyte invasion-associated gene expression signature

**DOI:** 10.1186/ar2476

**Published:** 2008-08-15

**Authors:** Teresina Laragione, Max Brenner, Wentian Li, Pércio S Gulko

**Affiliations:** 1 Laboratory of Experimental Rheumatology, Center for Genomics and Human Genetics, Feinstein Institute for Medical Research, 350 Community Drive, Manhasset, New York 11030, USA; 2 Genomics and Human Genetics, Feinstein Institute for Medical Research, 350 Community Drive Manhasset, New York 11030, USA; 3 Department of Medicine, New York University School of Medicine, 550 First Avenue, New York, 10016, USA

## Abstract

**Introduction:**

The *in vitro *invasive properties of rheumatoid arthritis (RA) fibroblast-like synoviocytes (FLSs) have been shown to correlate with disease severity and radiographic damage. We recently determined that FLSs obtained from pristane-induced arthritis (PIA)-susceptible DA rats are also highly invasive in the same *in vitro *assay through Matrigel. The transfer of alleles derived from the arthritis-resistant F344 strain at the arthritis severity locus *Cia5d *(RNO10), as in DA.F344(Cia5d) congenics, was enough to significantly and specifically reduce the invasive properties of FLSs. This genetically controlled difference in FLS invasion involves increased production of soluble membrane-type 1 matrix metalloproteinase (MMP) by DA, and is dependent on increased activation of MMP-2. In the present study we aimed to characterize the pattern of gene expression that correlates with differences in invasion in order to identify pathways regulated by the *Cia5d *locus.

**Methods:**

Synovial tissues were collected from DA and DA.F344(Cia5d) rats 21 days after the induction of PIA. Tissues were digested and FLSs isolated. After a minimum of four passages, FLSs were plated on Matrigel-covered dishes at similar densities, followed by RNA extraction. Illumina RatRef-12 expression BeadChip arrays were used. Expression data were normalized, followed by *t*-test, logistic regression, and cluster analysis. Real-time PCR was used to validate the microarray data.

**Results:**

Out of the 22,523 RefSeq gene probes present in the array, 7,665 genes were expressed by the FLSs. The expression of 66 genes was significantly different between the DA and DA.F344(Cia5d) FLSs (*P *< 0.01). Nineteen of the 66 differentially expressed genes (28.7%) are involved in the regulation of cell cycle progression or cancer-associated phenotypes, such as invasion and contact inhibition. These included *Cxcl10*, *Vil2 *and *Nras*, three genes that are upregulated in DA and known to regulate MMP-2 expression and activation. Nine of the 66 genes (13.6%) are involved in the regulation of estrogen receptor signaling or transcription. Five candidate genes located within the *Cia5d *interval were also differentially expressed.

**Conclusions:**

We have identified a novel FLS invasion associated gene expression signature that is regulated by *Cia5d*. Many of the genes found to be differentially expressed were previously implicated in cancer cell phenotypes, including invasion. This suggests a parallel in the behavior of arthritis FLSs and cancer cells, and identifies novel pathways and genes for therapeutic intervention and prognostication.

## Introduction

Rheumatoid arthritis (RA) is a common chronic autoimmune disease that affects approximately 1% of the population [1]. It is a complex trait, in which genetic and environmental factors mediate disease susceptibility and severity [1]. Basic joint pathology in RA is characterized by pronounced synovial hyperplasia, also called 'pannus', which produces several proinflammatory cytokines and proteases and, like a malignant tumor, invades and destroys cartilage and bone [2-4].

The formation of the synovial pannus is regulated by complex interactions between synovial resident cells and infiltrating inflammatory cells [5,6], and their production of paracrine and autocrine factors such as cytokines and growth factors [7-9], nuclear factor-kB activation [10], and angiogenesis [11]. The fibroblast-like synoviocyte (FLS) is a key player in this process, and its numbers are markedly increased in the hyperplastic synovial pannus of RA and rodent models of arthritis [4]. RA FLSs invade cartilage [12] and produce increased amounts of several proteolytic enzymes that further contribute to joint destruction [2,3]. The invasive properties of RA FLSs have also been associated with radiographic damage in RA, a parameter of disease severity, which emphasizes their direct clinical relevance [13].

We have previously identified *Cia5d *as an arthritis severity locus and showed that DA.F344(Cia5d) rats congenic for this interval developed significantly milder arthritis, with nearly no pannus formation and neither bone nor cartilage destruction, as compared with highly susceptible DA rats [14]. We also determined that *Cia5d *regulates the invasive properties of FLSs, thus providing an explanation for its role in joint damage [15]. The arthritis gene located within *Cia5d *controls the FLS production of soluble membrane-type 1 (MT1)-matrix metalloproteinase (MMP) and activation of MMP-2 [15]. This was the first time that FLS phenotypes were found to be genetically regulated.

In the present study we took advantage of this genetically regulated FLS invasive phenotype and compared highly invasive with minimally invasive cells' gene expression signatures using microarrays. The study of more than 22,000 genes identified a gene expression signature related to invasion that is differentially regulated between FLSs from DA and DA.F344(Cia5d) rats. The novel FLS invasion pathways described here resemble those described in cancer cell lines and have the potential to become novel targets for therapeutic intervention.

## Materials and methods

### Rats

**Figure 1:**
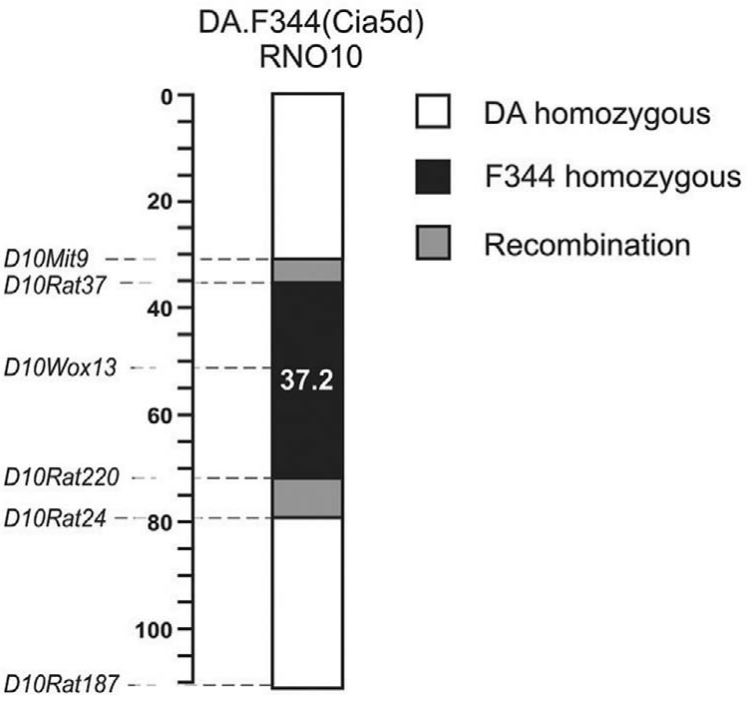
Map of *Cia5d *congenic interval. Markers used in the breeding of DA.F344(Cia5d) congenics and their positions on chromosome 10. Numbers represent the position in the chromosomes. Mb, megabases.

DA (DA/BklArbNsi, arthritis-susceptible) inbred rats (originally from Bentin & Kingman, CA, USA) were maintained at the Arthritis and Rheumatism Branch (Arb; National Institutes of Health) and then transferred to the Feinstein Institute (previously named North Shore-LIJ Institute; Nsi). The genotype-guided breeding of DA.F344(Cia5d) was previously described in detail [14]. Briefly, a 37.2 megabase interval on rat chromosome 10 was transferred from F344 into the DA background over 10 backcrosses followed by at least five intercrosses (Figure 1). The experiments were conducted with rats homozygous at the congenic interval. All experiments involving animals were reviewed and approved by the Feinstein Institute for Medical Research Institutional Animal Care and Use Committee. Animals were housed in a pathogen free environment, on 12-hour light and dark cycles, with free access to food and water.

### Induction of PIA and arthritis scoring

Rats aged 8 to 12 weeks received 150 μl of pristane by intradermal injection divided into two sites at the base of the tail [14,16]. The animals were scored on days 14, 18 and 21 after pristane induction using a previously described arthritis scoring system [17,18]. On day 21 after injection, the animals were killed and synovial tissue was collected from the ankles for FLS isolation.

### Isolation and culture of primary FLS

FLSs were isolated by enzymatic digestion of the synovial tissue. Briefly, tissues were minced and incubated with a solution containing DNase 0.15 mg/ml, hyaluronidase type I-S 0.15 mg/ml, and collagenase type IA 1 mg/ml (Sigma-Aldrich, St. Louis, MO, USA) in Dulbecco's modified Eagle's medium (DMEM; Gibco, Invitrogen Corporation, Carlsbad, CA, USA) for 1 hour at 37°C. Cells were washed and re-suspended in DMEM supplemented with 10% fetal bovine serum (Gibco), glutamine 30 mg/ml, amphotericin B 250 μg/ml (Sigma), and gentamicin 10 mg/ml (Gibco). After overnight culture, nonadherent cells were removed and adherent cells were cultured. All experiments were performed with cells after passage four (95% FLS purity).

### Flow-cytometric characterization of FLSs

Freshly trypsinized FLSs (105) were re-suspended in phosphate-buffered saline with 0.02% azide (Sigma-Aldrich) and 1% bovine serum albumin (P Biomedicals, Aurora, OH, USA), and incubated with 1 μg anti-CD32 (Pharmingen, San Diego, CA, USA) to block Fcγ II receptors. Cells were stained with saturating concentrations of CD90 (OX-7; PerCP, Pharmingen) or isotype control. Stained cells were fixed with 1% paraformaldehyde in phosphate-buffered saline and analyzed by flow cytometry in a FACSCalibur (Becton Dickinson, Franklin Lakes, NJ, USA), using the BD Cell-Quest™ Pro version 4.0.1 software (Becton Dickinson).

### FLS culture on Matrigel

We previously studied the invasive properties of FLSs through a collagen matrix (Matrigel). Cell interactions with the extracellular matrix are known to influence the expression of several genes, including activation of MMP-2 [19], which is a key mediator of the FLS invasive phenotype. Therefore, in order to study the gene expression signature of highly invasive and minimally invasive FLSs, cells were cultured under the same conditions as used in the invasion studies. Specifically, 100% confluent 75 cm^2 ^FLS culture flasks were trypsinized (trypsin 0.25% with EDTA 0.1%). The rates of cellular proliferation differed among cell lines, and we previously showed that FLS proliferation does not correlate with the FLS invasive behavior. In order to have similar cell confluence at the time of FLS harvesting for RNA extraction, 10% to 50% of the high-density 75 cm^2 ^cell culture flasks (depending on the cell line) were plated in Matrigel-coated 10 cm culture dishes (Becton Dickinson) with DMEM, 10% fetal bovine serum, antibiotics, and glutamine. Cell cultures were maintained at 37°C with 5% carbon dioxide for 24 hours. After 24 hours, FLSs were harvested using a cell scraper (Corning, Acton, MA, USA) followed by digestion of the Matrigel with 10 ml collagenase D 1 mg/ml (Roche Applied Science, Indianapolis, IN, USA) at 37°C for 10 minutes. FLSs were then collected by centrifugation, washed twice with ice-cold phosphate-buffered saline. Cell pellets were re-suspended in RLT lysis buffer (RNeasy Mini Kit; Qiagen, Valencia, CA, USA) with 1% (vol/vol) β-mercaptoethanol (Sigma). Cell-lysis buffer suspension was vortexed, frozen in liquid nitrogen and stored at -80°C until RNA extraction.

### RNA extraction and quality assessment

Cells in RLT buffer were disrupted using QIAshredder spin columns (Qiagen), and total RNA was extracted using the RNeasy Mini Kit (Qiagen), in accordance with the manufacturer's instructions. Samples were digested with DNase (Qiagen) and eluted with 30 μl RNase-free water. RNAs were quantified and assessed for purity using a NanoDrop spectrophotometer (Rockland, DE, USA). RNA integrity was verified with a BioAnalyzer 2100 (Agilent, Palo Alto, CA, USA).

### RNA preparation and microarray experiments

The RatRef-12 Expression BeadChip contains 22,524 probes for a total of 22,228 rat genes selected primarily from the NCBI RefSeq database (Release 16; Illumina, San Diego, CA, USA), and was used in accordance with the manufacturer's instructions. All reagents have been optimized for use with Illumina's Whole-Genome Expression platform. Total RNA 200 ng was used for cRNA *in vitro *transcription and labeling with the TotalPrep™ RNA Labeling Kit using Biotinylated-UTP (Ambion, Austin, TX, USA). Hybridization is carried out in Illumina Intellihyb chambers at 58°C for 18.5 hours, which is followed by washing and staining, in accordance with the Illumina Hybridization System Manual. The signal was developed by staining with Cy3-streptavidin. The BeadChip was scanned on a high resolution Illumina BeadArray reader, using a two-channel, 0.8 μm resolution confocal laser scanner.

### Data extraction and normalization

The Illumina BeadStudio software (Version 2.0) was used to extract and normalize the expression data (fluorescence intensities) for the mean intensity of all 12 arrays. Genes expressed in all 12 arrays were selected for analyses. Normalized data were analyzed using the *t*-test and logistic regression.

### Statistics and analyses

The *t*-test was used to compare means of the log-transformed and non-log-transformed data. Genes with a *P *value under 0.01 between DA and DA.F344(Cia5d) were considered significant and included in additional analysis. The logistic regression model fitting was carried out as previously described [20,21] using the filtered gene list. The statistical significance of a logistic regression result was obtained by comparing the deviance with the 'null deviance'. This null deviance is the (-2)log-likelihood of a random model in which the probability for a sample to belong to a group (for example, DA) is equal to the proportion of DA samples in the dataset. The difference between the deviance and the null deviance follows the χ^2 ^distribution with one degree of freedom by chance alone, and this χ^2 ^distribution was used to determine the *P *value. The R statistical package [22] was used for *t*-test and logistic regression analyses.

The Ingenuity IPA 5.5.1 program (Ingenuity, Redwood City, CA, USA) and PubMed and GEO (Gene Expression Omnibus) searches were used for pathways detection. CLUSTER [23] and TREEVIEW [24] were used for cluster analysis and generation of a heat map.

### Quantitative real-time PCR

The same RNA used for the microarray experiments was also used for the quantitative real-time PCR confirmation experiments. Total RNA 200 ng from each sample was used for cDNA synthesis using the Superscript III kit (Invitrogen). Primers and probe sequences were designed to target the same exon as used in the Illumina RatRef-12 Expression BeadChip. We used Exiqon (Woburn, MA, USA) and Taqman (ABI, Applied Biosystems, Foster City, CA) probes (Table 1). GAPDH was used as endogenous control. Probes were labeled with FAM at the 5' end and TAMRA at 3' end and used at a final concentration of 100 nmol/l. Primers were used at 200 nmol/l concentration with Eurogentec quantitative real-time PCR mastermix (Eurogentec, San Diego, CA, USA). The ABI 7700 quantitative real-time PCR thermocycler was used at 48°C for 30 minutes, 95°C for 10 minutes, and 45 cycles of 95°C for 0.15 minutes and 60°C for 1 minute. Samples were run in duplicates and the means used for analysis. Data were analyzed using Sequence Detection System software version 1.9.1 (ABI). Results were obtained as Ct (threshold cycle) values. Relative expression of all the genes was adjusted for GAPDH in each sample (ΔCt), and ΔCt used for *t*-test analysis. Quantitative real-time PCR fold differences were calculated with 2^-ΔΔCt ^[25].

**Table 1:** Genes studied with QPCR for confirmatory studies, primers and probe sequences

**Accession number**	**Gene symbol**	**Target exon^b^**	**Probe**	**Forward primer**	**Reverse primer**
**Up-regulated in DA**					
NM_139089.1	Cxcl10	4	Exiqon Universal probe 67	TTCGGACCAGCTCTTAGAGAA	GCCTGGTCCTGAGACAAAAG
XM_220552.3	Trim16	6	Exiqon Universal probe 1	GTGAACTCCTTCCCACTCCA	CAGCTGCATTTCTGGAAACA
NM_017207.1	Trpv2	15	Exiqon Universal probe 6	CTCTTCCCACCTTATCTGAGGA	GACCTGAAGGGGCAGATG
NM_019357.1	Vil2	13	CCCCAAGACCCAGTGGAATCCTCC^a^	AGGTACCGGGCGATGTTCT	GGCCTGTTTGGCACTATGTGA
LOC309362	Dnmbp	16	Exiqon Universal probe 97	TTGTCTCAGCATGGGTCCTA	ACCAGGATTTTAAGGCCACA
NM_001107408	Gins3	3–4	Exiqon Universal probe 17	GTCGTGGACCTCCACAAAAT	GAACCGTCCAATAAAAGTCTGC
**Down-regulated in DA**					
XM_235434.4	Gsdmdc1	13	Exiqon Universal probe 68	AGCACGTCTTGGAACAGAGC	TCCTCATCCCAGCTGTCC
XM_222868.4	Olfml2b	8	Exiqon Universal probe 106	CTCCCTTCTTCCATGCTCTG	GCAAGCCCCAGAGGAATAA
NM_001008321.1	Gadd45b	4	Exiqon Universal probe 25	ACAGGTGGTCGCCAAGAC	CCAGGCCTTGGCTCTAAAGT
**Estrogen receptors**					
NM_012689.1	Esr1	-	Exiqon Universal probe 67	GCAAGAATGTCGTGCCTCTC	TGAAGACGATGAGCATCCAG
NM_012754	Esr2	-	Exiqon Universal probe 94	CCTTGAAGGCTCTCGGTGTA	CAGAACCTTTCAGATGTTTCCA

^a^Taqman probe. ^b^Same region used in the Illumina microarray.

## Results

### Characterization of the FLS cell lines used

In previous studies we determined that DA FLSs were highly invasive, and that alleles derived from the arthritis-resistant strain F344 at the *Cia5d *interval, as in DA.F344(Cia5d) congenics (Figure 1), specifically reduced the invasive properties of FLSs. Additionally, FLSs from DA and DA.F344(Cia5d) strains expressed similar mRNA levels of transforming growth factor-β, tumor necrosis factor-α, IL-1β and IL-6, as well as MMP-1, MMP-2, MMP-3, MMP-9, MMP-13, MT1-MMP and MT2-MMP [15]. Both strains had similar collagenase and MMP-3 activity, but levels of soluble MT1-MMP and active MMP-2 were increased in DA. MMP-2 inhibition reduced DA FLS invasion to levels similar to those of DA.F344(Cia5d). Cytoskeleton characteristics were also similar in DA and DA.F344(Cia5d) FLSs [15].

In the present study FLSs were stained with CD90, a marker for FLS [26], and analyzed by flow cytometry. Comparable numbers of CD90^+ ^cells were detected both in five different DA and five different DA.F344(Cia5d) rats (percentage of CD90^+ ^cells [mean ± standard deviation]: DA 95.46 ± 8.9 and DA.F344 [Cia5d] 96.51 ± 5.9), demonstrating that the cell lines were homogeneously CD90^+^.

### Genes expressed by FLSs and filtering criteria

A total of 7,665 genes out of 22,228 genes represented in the Illumina RatRef-12 BeadChip were expressed by both DA and DA.F344(Cia5d) FLSs. Log transformation did not significantly affect the list of differentially expressed genes, and therefore results are shown from analyses done with non-log-transformed data.

### Genes differentially expressed between DA and DA.F344(Cia5d) FLSs

**Figure 2:**
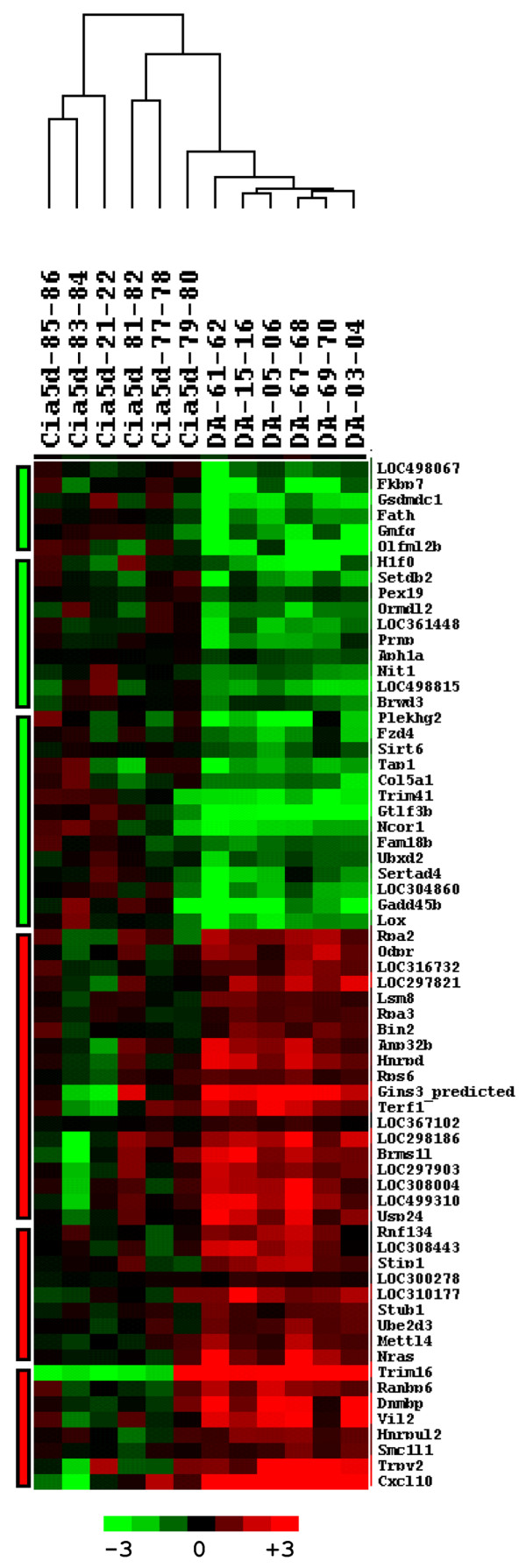
Cluster analysis and heat map of 66 differentially expressed genes. DA and DA.F344(Cia5d) samples are clustered on columns and genes on rows. Bars on the left side of the figure identify the three clusters of genes with reduced expression (green) and the three clusters of genes with increased expression (red) in DA compared with DA.F344(Cia5d).

Sixty-six genes had a *P *value under 0.01 (Tables 2 and 3) and were used for fold change calculations and pathway detection analyses. Thirty-six genes were expressed in increased levels by DA FLSs, and the presence of F344 alleles at the *Cia5d *interval, as in DA.F344(Cia5d) congenics FLSs, was enough to reduce their expression significantly (Table 2). Thirty genes were expressed in reduced levels in DA and significantly increased in DA.F344(Cia5d) FLSs (Table 3). These observations demonstrate that alleles within the *Cia5d *interval, the only genetic difference between DA and DA.F344(Cia5d), are directly or indirectly involved in the regulation of the expression of several genes, and the difference in gene expression correlates with the difference in invasive properties of FLSs. Furthermore, cluster analysis separated DA FLSs from DA.F344(Cia5d) FLSs, demonstrating that the two strains could be reliably differentiated by gene expression (Figure 2).

**Table 2:** Genes with reduced expression in synovial fibroblasts from DA.F344 (Cia5d) compared with highly invasive DA, including those associated with cancer-phenotypes and estrogen signaling

**Gene Symbol^d^**	**Definition^a^**	**Accession number**	**DA mean**	**Cia5d mean**	**Fold change**	**P value^b^**	**Overall rank^c^**
**Cancer, Cell Cycle, DNA replication, recombination and repair**							
***Trim16_predicted* ^e^**	**Tripartite motif protein 16 (predicted)**	XM_220552.3	262.14	82.27	-3.2	0.0033	23
***Cxcl10***	**Chemokine (C-X-C motif) ligand 10^f^**	NM_139089.1	1218.54	434.48	-2.8	0.0001	2
*Dnmbp*	Similar to Dynamin binding protein (Scaffold protein Tuba)	XM_219860.3	739.97	385.61	-1.9	0.0088	62
***Vil2***	**Villin 2 (Ezrin)^f^**	NM_019357.1	1642.95	984.09	-1.7	0.0023	15
*Nras*	Neuroblastoma RAS viral (v-ras) oncogene homolog^f^	XM_579607.1	910.25	601.06	-1.5	0.0087	60
*Brms1l_predicted*	Breast cancer metastasis-suppressor 1-like (predicted)	XM_216712.3	187.93	125.37	-1.5	0.0094	64
*Hnrpd*^e^	Heterogeneous nuclear ribonucleoprotein D (AU-rich element RNA binding protein 1, 37 kDa)	NM_024404.1	2909.16	1959.49	-1.5	0.0010	8
*Rpa2*	Replication protein A2	NM_021582.1	1583.81	1154.73	-1.4	0.0074	48
*Ube2d3*	Ubiquitin-conjugating enzyme E2D 3	NM_031237.1	123.48	99.45	-1.2	0.0017	10
*Lsm8_predicted*^e^	LSM8 homolog, U6 small nuclear RNA associated (S. cerevisiae) (predicted)	XM_216102.3	3766.75	3121.49	-1.2	0.0024	16
*Smc1l1*	Structural maintenance of chromosomes 1 like 1 (S. cerevisiae)	NM_031683.1	4648.45	3923.73	-1.2	0.0044	30
*Rpa3_predicted*	Replication protein A3 (predicted)	XM_216097.3	4013.83	3410.52	-1.2	0.0022	14
							
**Cell Signaling**							
**Stip1**	**Stress-induced phosphoprotein 1 (Stip1)**	NM_138911.2	3478.09	2568.75	-1.4	0.0028	18
							
**Ubiquitination**							
Usp24_predicted	Ubiquitin specific protease 24 (predicted)	XM_233260.3	111.07	74.14	-1.5	0.0037	25
**Stub1_predicted**	**STIP1 homology and U-Box containing protein 1 (predicted)**	XM_213270.3	4967.20	4164.69	-1.2	0.0034	24
							
**Ribosomal Proteins**							
Rps6	Ribosomal protein S6 (Rps6)	NM_017160.1	29305.46	24538.18	-1.2	0.0085	57
LOC300278	Similar to 40S ribosomal protein S9	XM_213106.3	28115.69	26209.24	-1.1	0.0086	59
LOC367102	Similar to 40S ribosomal protein S9	XM_345948.2	25678.47	23353.32	-1.1	0.0043	28
							
**Others**							
Trpv2	Transient receptor potential cation channel, subfamily V, member 2	NM_017207.1	177.90	92.25	-1.9	0.0075	49
**Gins3_predicted^e^**	**GINS complex subunit 3 (Psf3 homolog) **	XM_226235.2	171.57	89.64	-1.9	0.0010	6
LOC499310	Similar to cell division cycle associated 5	XM_574612.1	450.69	270.81	-1.7	0.0061	44
LOC298186	Similar to hypothetical protein FLJ33868 (predicted)	XM_238399.3	271.10	177.29	-1.5	0.0070	46
Terf1_predicted	Telomeric repeat binding factor 1 (predicted)	XM_238387.3	98.95	66.02	-1.5	0.0048	34
LOC308004	Similar to hypothetical protein FLJ13188 (predicted)	XM_217663.3	573.01	383.19	-1.5	0.0083	56
LOC310177	Similar to RIKEN cDNA 0610040D20	XM_226872.2	85.32	58.03	-1.5	0.0044	29
LOC297821	Similar to F23N19.9 (predicted)	XM_232684.3	1680.52	1185.76	-1.4	0.0052	36
LOC308443	Similar to CDNA sequence BC028440	XM_218345.2	426.63	301.59	-1.4	0.0059	41
Anp32b	Acidic nuclear phosphoprotein 32 family, member B	NM_131911.2	454.58	323.06	-1.4	0.0082	55
Ranbp6_predicted	RAN binding protein 6 (predicted)	XM_219796.2	309.74	222.79	-1.4	0.0031	22
LOC297903	Similar to RIKEN cDNA 6720467C03 (predicted)	XM_216357.3	1493.92	1088.11	-1.4	0.0075	50
Qdpr	Quinoid dihydropteridine reductase	NM_022390.1	983.32	728.72	-1.3	0.0045	33
Rnf134_predicted	Ring finger protein 134 (predicted)	XM_219963.3	952.04	717.85	-1.3	0.0059	42
LOC316731	Similar to hypothetical protein FLJ23017 (predicted)	XM_237515.3	74.86	58.48	-1.3	0.0094	65
LOC309197	Similar to hypothetical protein	XM_219560.3	1413.35	1112.64	-1.3	0.0050	35
LOC316732	Similar to RIKEN cDNA 4931400A14 (predicted)	XM_244261.3	251.40	201.41	-1.2	0.0062	45
Bin2_predicted	Bridging integrator 2 (predicted)	XM_578696.1	57.42	47.13	-1.2	0.0076	51

^a^Estrogen; ER, estrogen-induced, or estrogen-receptor signaling or degradation are marked in bold. ^b^t test. ^c^Order (logistic regression) in the list of 66 genes differentially expressed between DA and DA.F344(Cia5d). ^d^Cancer and invasion associated genes are in italics. ^e^Differentially expressed in prostate, breast, colon or pharyngeal cancers. ^f^Known to induce transcription or activation of gelatinases.

**Table 3:** Genes with increased expression in synovial fibroblasts from DA.F344 (Cia5d) compared with DA

**Gene Symbol^d^**	**Definition^a^**	**Accession number**	**DA mean**	**Cia5d mean**	**Fold change**	**P value^b^**	**Overall rank^c^**
**Cancer, Cell Cycle, DNA replication, recombination and repair**							
***Gadd45b***	**Growth arrest and DNA-damage-inducible 45 beta **	NM_001008321.1	214.12	412.97	1.9	0.00572	39
***Gmfg***	**Glia maturation factor, gamma (Gmfg)**	NM_181091.2	1359.39	2261.87	1.7	0.00817	54
*Plekhg2_predicted*	Pleckstrin homology domain containing, family G (with RhoGef domain) member 2 (predicted)	XM_214862.3	91.97	147.62	1.6	0.00784	52
*Lox*	Lysyl oxidase	XM_579391.1	15755.11	24559.79	1.6	0.00198	12
*Brwd3_predicted*	Similar to bromo domain-containing protein disrupted in leukemia (LOC317213)	XM_228518.3	43.85	52.99	1.2	0.00596	43
*Aph1a*	Similar to anterior pharynx defective 1 homolog A (C. elegans)	XM_345251.2	2820.66	3246.28	1.2	0.00046	4
*Pex19_predicted*^e^	Peroxisome biogenesis factor 19 (predicted)	XM_225711.3	119.41	135.98	1.1	0.00561	38
							
**Cell Signaling**							
Fkbp7_predicted	FK506 binding protein 7 (predicted)	XM_215758.3	784.02	1450.53	1.9	0.00578	40
**Ncor1**	**Nuclear receptor co-repressor 1**	XM_577103.1	420.35	679.65	1.6	0.00454	32
Tap1	Transporter 1, ATP-binding cassette, sub-family B (MDR/TAP)	NM_032055.1	190.32	288.49	1.5	0.00878	61
Prnp	Prion protein	XM_579340.1	17242.89	24050.29	1.4	0.00029	3
Fzd4	Frizzled homolog 4 (Drosophila)	NM_022623.1	44.45	60.14	1.4	0.00406	26
							
**Gene expression**							
H1f0	H1 histone family, member 0	NM_012578.2	150.12	229.40	1.5	0.00707	47
**Cell-Cell Interaction**							
Fath	Hypothetical gene supported by NM_031819; Fath fat tumor suppressor homolog (Drosophila)	XM_579538.1	3803.04	5806.86	1.5	0.00206	13
							
**Extracellular Matrix**							
Col5a1	Collagen, type V, alpha 1 (Col5a1)	NM_134452.1	7240.26	9852.22	1.4	0.00807	53
							
**Others**							
Gtlf3b_predicted	Gene trap locus F3b (predicted)	XM_343907.2	78.16	175.41	2.2	0.00003	1
Olfml2b_predicted	Olfactomedin-like 2B (predicted)	XM_222868.3	1336.30	2949.43	2.2	0.00241	17
Gsdmdc1_predicted	Gasdermin domain containing 1 (predicted)	XM_235434.3	458.74	831.39	1.8	0.00295	20
Trim41_predicted	Tripartite motif-containing 41 (predicted)	XM_220357.3	422.66	732.37	1.7	0.00100	7
LOC498815	Hypothetical gene supported by AY771707	XM_579873.1	243.56	366.68	1.5	0.00281	19
LOC304860	Similar to N-acetylneuraminate pyruvate lyase	XM_222736.3	270.64	401.65	1.5	0.00176	11
Setdb2_predicted	SET domain, bifurcated 2 (predicted)	XM_224248.3	94.38	136.31	1.4	0.00945	66
LOC361448	Similar to cDNA sequence BC013529 (predicted)	XM_341726.2	2852.12	4043.46	1.4	0.00071	5
LOC360899	Similar to SERTA domain containing 4	XM_341174.2	1771.29	2489.20	1.4	0.00886	63
Ormdl2_predicted	ORM1-like 2 (S. cerevisiae) (predicted)	XM_213832.3	1996.56	2773.15	1.4	0.00549	37
LOC498067	Similar to RIKEN cDNA 2310003P10 (LOC498067), mRNA.	XM_573266.1	368.00	494.10	1.3	0.00860	58
Nit1	Nitrilase 1	NM_182668.1	3397.58	4472.84	1.3	0.00296	21
Fam18b_predicted	Family with sequence similarity 18, member B (predicted)	XM_219680.3	2915.92	3746.20	1.3	0.00447	31
Ubxd2_predicted	UBX domain containing 2 (predicted)	XM_573443.1	2018.75	2569.23	1.3	0.00411	27

^a^Estrogen; ER, estrogen-induced, or estrogen-receptor signaling or degradation are marked in bold. ^b^t test. ^c^Order (logistic regression) in the list of 66 genes differentially expressed between DA and DA.F344(Cia5d). ^d^Cancer and invasion associated genes are in italics. ^e^Increased expression in invading breast cancers.

### Genes upregulated in the highly invasive DA FLSs and downregulated in DA.F344(Cia5d) include cancer-associated and invasion regulatory genes

**Figure 3:**
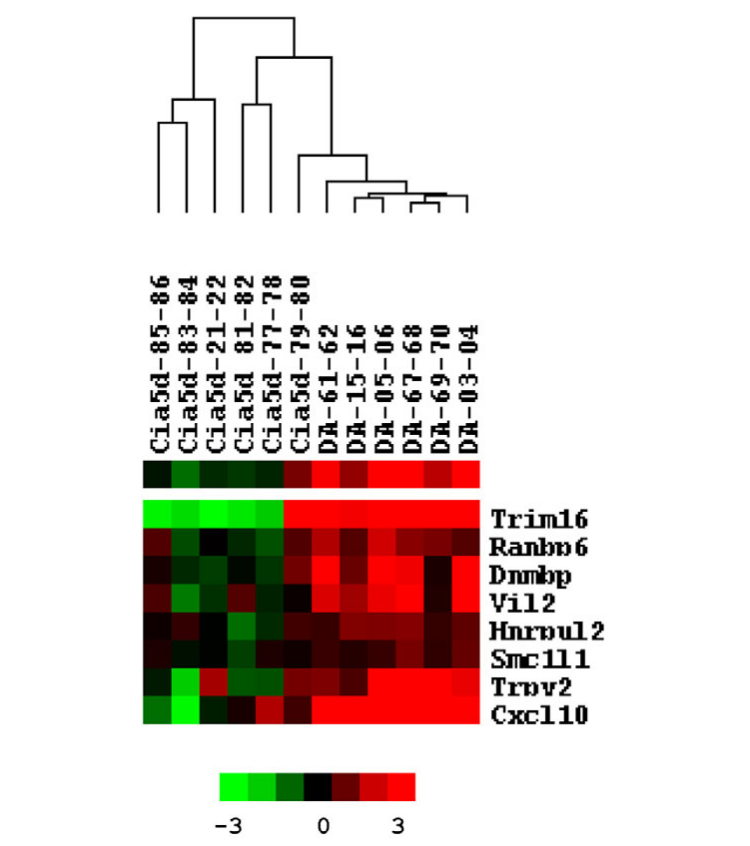
Cluster containing invasion and cancer-associated genes. Detailed view of the cluster that contains genes implicated in invasion and other cancer-associated phenotypes, including *Cxcl10, Vil2 *and *Dnmbp*.

Cluster analysis identified three main clusters among the genes expressed in increased levels in DA (Figure 2). One of the three clusters contained eight genes, three of which have been implicated in cancer and cancer-related cellular phenotypes such as invasion, and included *Cxcl10*, *Vil2 *and *Dnmbp *(Figure 3). The other genes in this cluster are involved in ion transport (*Trpv2*), mitosis (*Smc1L1*), or have incompletely characterized functions (*Trim16*, *Ranbp6 *and *Hnrpul2*). In total, 12 out of the 36 genes (33.3%) expressed in increased levels by DA FLSs and downregulated in DA.F344(Cia5d) are known to regulate cancer-associated processes, including cell cycle progression (*Rpa2 *and *Rpa3*), cell invasion (*Cxcl10*, *Vil2*, *Nras*, and *Dnmbp*), and metastasis (*Vil2 *and *Brms1l*), respectively (Table 2). In fact, *Cxcl10 *was the second best discriminator between DA and DA.F344(Cia5d) cell lines, as per logistic regression (Table 2).

**Figure 4:**
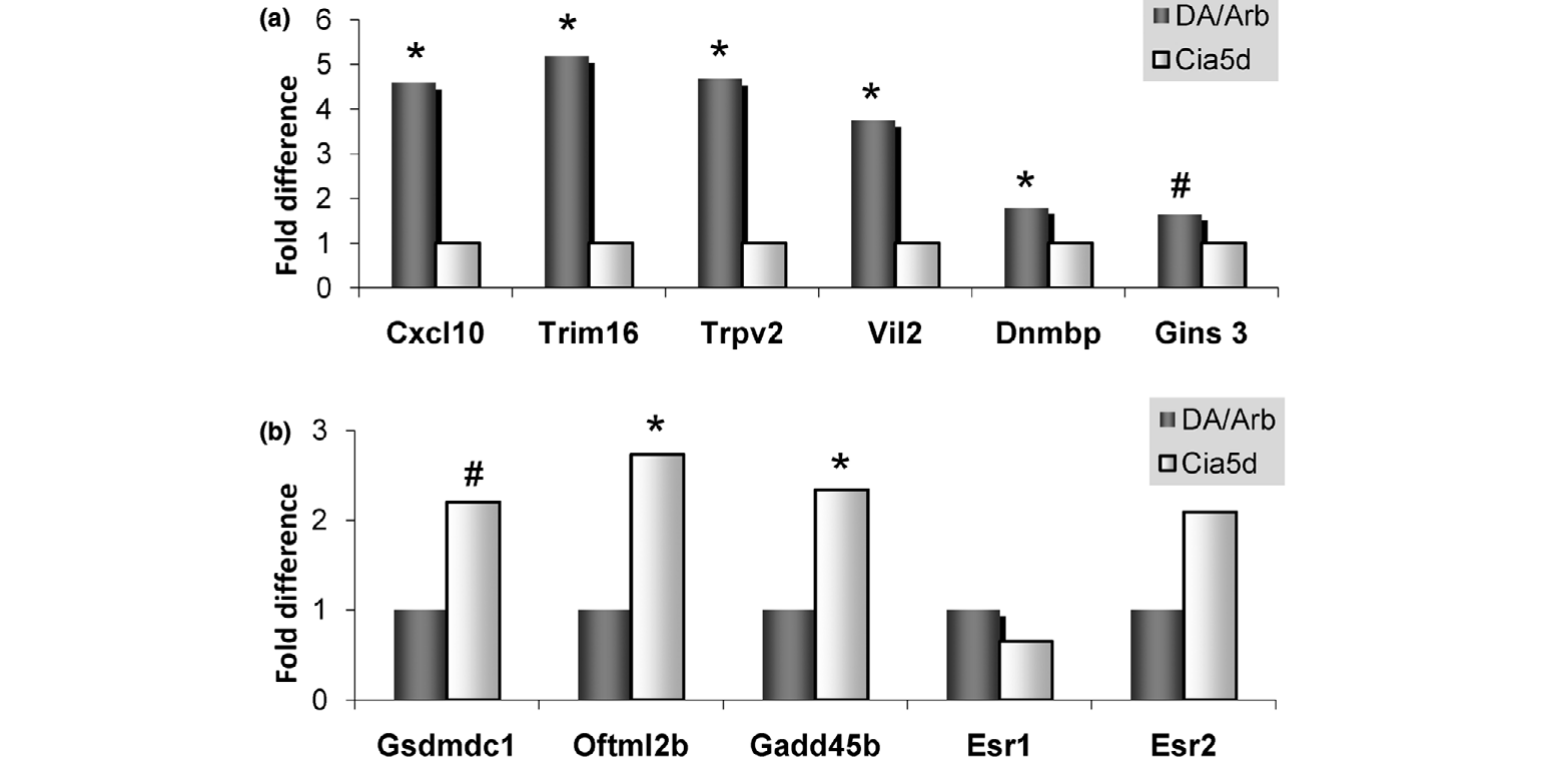
Quantitative real-time PCR. Presented are quantitative real-time PCR analysis of **(a) **genes upregulated in DA and downregulated in DA.F344(Cia5d), and **(b) **genes downregulated in DA and upregulated in DA.F344(Cia5d). These include invasion and cancer-associated genes and estrogen-inducible genes. Estrogen receptors *Esr1 *and *Esr2 *were also analyzed. *P < 0.05, ^#^*P *< 0.07.

Of additional interest in relation to the MMP-2-dependent difference in FLS invasion that we have observed, three of these genes – namely *Cxcl10*, *Vil2 *and *Nras *– are known to regulate the synthesis or activation of gelatinases. Increased levels of *Cxcl10*, *Vil2*, *Dnmbp*, *Trim16*, and *Trpv2 *in DA were confirmed using quantitative real-time PCR, with most of these genes having a nearly fourfold or greater difference in expression (*P *< 0.05; Figure 4a).

### Genes downregulated in the highly invasive DA FLSs and upregulated in DA.F344(Cia5d) include tumor suppressor and cell cycle check-point genes

The list of genes with reduced expression in DA, as compared with increased expression in DA.F344(Cia5d) congenics, included seven genes that are involved in tumor suppression-like activity and cell cycle check-points, such as *Aph1a*, *Brwd3*, *Gadd45b*, *Gmfg*, *Lox*, and *Plekhg2 *(Table 3). *Gadd45b *was chosen for quantitative real-time PCR confirmation (*P *< 0.05; Figure 4b). These observations, combined with the 11 cancer and invasion associated genes upregulated in DA, suggest an invasion-favoring profile similar to that described in cancer cells, characterized by reduced expression cell cycle check-point and tumor suppressor genes combined with increased expression of invasion genes.

### Additional genes with reduced expression in DA FLSs

Additionally, *Ubxd2*, *Fzd4*, *Fkbp7*, *Olfml2b*, *Gsdmdc1 *and the transcriptional co-repressor *Ncor1 *were among the genes downregulated in DA and with increased expression in DA.F344(Cia5d). *Gtlf3b *(predicted), a gene trap fragment with unknown function, was among the most significantly differentially expressed genes (*P *= 0.000025; 2.2-fold difference; Table 3). The greater than twofold difference in expression of *Olfml2b *and *Gsdmdc1 *was confirmed with quantitative real-time PCR (Figure 4b).

### Increased number of estrogen-inducible and ER signaling regulatory genes among the differentially expressed genes

Nine genes or 13.6% of the 66 differentially expressed genes were either estrogen-inducible genes, such as *Cxcl10*, *Vil2*, *Trim16*, *Gins3 *(predicted), and *Gadd45b*, or genes involved in modulating the estrogen receptor (ER) signaling such as *Stub1 *and *Stip1*. *Ncor1 *negatively regulates ER-mediated transcription and its levels were also reduced in DA, further suggesting unopposed ER-mediated transcription. The differential expression of *Cxcl10*, *Vil2*, *Trim16*, *Gins3*, and *Gadd45b *was confirmed with quantitative real-time PCR (Figure 4a, b). The ERs *Esr1 *and *Esr2 *were not differentially expressed in the microarray analysis, and those results were confirmed with quantitative real-time PCR (Figure 4b). There was a trend toward increased expression *Esr2 *in DA.F344(Cia5d), but that difference did not reach statistical significance (*P *= 0.093; Figure 4b). Taken together, this pattern of gene expression suggests that the invasive DA FLSs have an enhanced ER activity regulated at different levels that could include reduced degradation of the ER, reduced inhibition of the ER-mediated transcription, and increased levels of estrogen-inducible genes.

### Five of the differentially expressed genes are located within the Cia5d interval

Five out of the 66 differentially expressed genes were located within the *Cia5d *interval (Table 4). The number of genes located within the *Cia5d *interval found to be differentially expressed between DA and DA.F344(Cia5d) FLSs was greater than would be expected by chance (3.3% observed versus 0.8% expected by chance; *P *= 0.0044 by χ^2 ^with Yates correction; Table 5).

**Table 4:** Differentially expressed genes located within the Cia5d interval on rat chromosome 10

**Symbol**	**Definition**	**Accession number**	**Position (Mb)**	**Cytogenetic**	**DA mean**	**Cia5d mean**	**Fold change**	**t test**	**Overall rank**
**Reduced levels in Cia5d**									
Trim16	Tripartite motif protein 16 (predicted) (Trim16_predicted)	XM_220552.3	48.95	10q23	262.14	82.27	-3.19	0.00326	23
Trpv2	Transient receptor potential cation channel, subfamily V, member 2 (Trpv2)	NM_017207.1	48.76	10q23	177.90	92.25	-1.93	0.00745	49
**Increased levels in Cia5d**									
Ncor1	Nuclear receptor co-repressor 1 (Ncor1)	XM_577103.1	48.62	10q23	420.35	679.65	1.62	0.00454	32
Gtlf3b	Gene trap locus F3b (predicted) (Gtlf3b_predicted)	XM_343907.2	47.05	10q22	78.16	175.41	2.24	0.00003	1
Trim41	Tripartite motif-containing 41 (predicted)	XM_220357.3	34.08	10q21	422.65	732.37	1.73	0.00099	7

**Table 5:** A greater than expected number of genes located within the Cia5d interval were differentially expressed in FLS^a^

	**Differentially expressed**	**Not-differentially expressed**
Genes located within Cia5d	5 (3.3%)	146
Genes located outside Cia5d	61 (0.8%)	7453

^a^p-value = 0.00442 (Chi-square with Yates correction).

*Trim16*, *Trpv2*, and *Ncor1 *are closely located on chromosome 10q23, raising the possibility that a polymorphism in a regulatory region or intron in this region, or even in one of these genes, could account for the difference in expression detected between the two strains.

## Discussion

RA histology is typically characterized by pronounced synovial hyperplasia, also called 'pannus'. The RA pannus produces proinflammatory cytokines and proteases, and invades cartilage and bone leading to joint destruction and deformities [4]. The FLS is a key player in RA pannus and joint pathology, and has increased invasive properties, compared with osteoarthritis, even after several passages *in vitro *[12,27]. Furthermore, the increased invasive properties of RA FLSs have been associated with increased radiographic joint destruction [13], underscoring the relevance of this *in vitro *phenotype to disease outcome.

We recently described the first evidence that the invasive properties of FLSs are genetically regulated [15]. We determined that a gene located within the arthritis severity regulatory *Cia5d *interval specifically controls the invasive properties of FLSs via the regulation of the production of soluble MT1-MMP and activation of MMP-2 [15]. Levels of active MMP-2 are also increased in the synovial fluid of patients with RA, and correlate with disease severity and radiographic damage [28]. Therefore, understanding the regulation of cell invasion and MMP-2 activation is highly relevant to RA. In addition, several common cancers have increased levels of MMP-2, which correlates with worse prognosis [29-36], suggesting that identifying the *Cia5d *gene and the pathways controlled by it could potentially generate novel targets relevant to cancer treatment as well.

In the present study we used a novel strategy to identify differences in gene expression that correlate with the invasive properties of FLSs. First, two closely related strains were used. These strains have identical DA genomes, except that DA.F344(Cia5d) congenics have F344 arthritis-resistant alleles in a 37.2 megabase interval on chromosome 10. This strategy minimized noise related to allelic variations at other regions of the genome that are not related to the phenotype of interest. Second, instead of using synovial tissues, which have mixed cellularities that interfere with the interpretation of the results, we generate and used primary FLS cell lines. Third, FLSs from DA and DA.F344(Cia5d) differ in their invasive properties, thus providing a more precise phenotype. Finally, the cells used for RNA extractions were cultured on the same collagen matrix (Matrigel) used in the invasion experiments, hence recreating the same *in vitro *environment. This latter aspect is critical because extracellular matrix and cell influence processes that are central to cell invasion, such as the expression of adhesion molecules and MMP-2 activation [19], and are required for proper activation of the invasive phenotype, including gene transcription. This strategy led to the identification of new genes involved in FLS invasion.

A genome-wide analysis of gene expression conducted with RA FLSs suggested two patterns that correlated with increased or reduced inflammation in the tissues of origin [37]. Those RA FLSs were not studied for invasion, and there was no control group without erosive changes for comparison. Furthermore, the RNA was obtained from cells cultured on plastic dishes and not on a collagen matrix such as Matrigel. Therefore, it was not surprising that using different methodologies to address a different question we detected a new FLS invasion signature that is different from the two RA FLS gene expression patterns previously reported.

A genome-wide microarray-based gene expression analysis was conducted to identify genes and pathways that are differentially expressed between highly invasive DA and minimally invasive DA.F344(Cia5d) FLSs. The analysis revealed that 66 genes out of the 7,665 genes expressed by FLSs were differentially expressed between DA and DA.F344(Cia5d) FLSs (*P *< 0.01). Nineteen of the 66 differentially expressed genes (28.7%) had previously been implicated in tumor suppression activity or other cancer cell phenotypes, but had not been implicated in the invasive properties of the FLSs. These cancer-related phenotypes include malignant transformation (*Hnrpd*) [38], tumor growth (*Ach1a *and *Gfmg*) [39,40], oncogene-like activity (*Plekgh2*) [41], tumor apoptosis (*Gadd45b*) [42], tumor suppressor activity (*Brwd3*) [43], cancer cell growth arrest (*Ube2d3*) [44], contact inhibition (*Gmfg*) [45], and cell invasion (*Lox, Ach1a*, *Cxcl10*, *Vil2*, and *Nras*) [46-50]. Genetic variations in DNA synthesis gene *Rpa3 *have been associated with susceptibility to carcinomas [51], whereas increased cancer expression of *Rpa2 *is associated with adverse outcome in colon cancer [52]. Some of these genes were found to be expressed in increased levels in certain cancers (*Hnrpd *and *Lsm8*) [53,54], including highly invasive types [55]. These observations suggest that FLSs derived from arthritis joints and cancer cells share common processes in the regulation of cell invasion, and that these processes are in part regulated by a gene located within the arthritis severity locus *Cia5d*.

**Figure 5:**
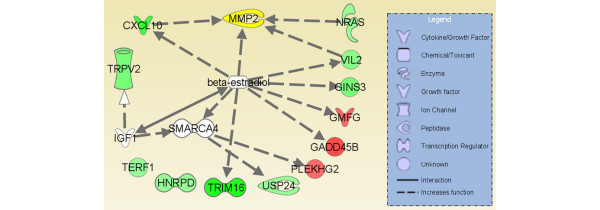
Known interactions between genes differentially expressed in FLSs from DA and DA.F344(Cia5d). Several estrogen (β-estradiol)-regulated genes, including genes involved in increasing either the expression or activity of matrix metalloproteinase (MMP)-2, such as *Cxcl10*, *Nras*, and *Vil2 *were differentially expressed. Green indicates downregulated in DA.F344(Cia5d) and upregulated in DA. Red indicates upregulated in DA.F344(Cia5d) and downregulated in DA. White indicates that the gene is part of the pathway but was not differentially expressed in fibroblast-like synoviocytes (FLSs) from the two strains. Color intensity correlates with magnitude of the expression difference.

*Nras *[56,57], *Vil2 *(encoding the ezrin protein) [49,50], and *Cxcl10 *[58] – three genes that are upregulated in DA but downregulated in DA.F344(Cia5d) – have also been implicated in the regulation of gelatinases' expression and activation, including MMP-2 (Figure 5). These observations provide a direct link between the invasion and MMP-2 phenotypes that we have been studying and the gene expression signature regulated by the *Cia5d *locus. Furthermore, studies with RA synovial tissues [59,60] and RA FLSs [60] have also demonstrated increased expression of *Cxcl10 *both at mRNA and protein levels. *Cxcl10 *has also been shown to increase the production and activity of gelatinases in RA FLSs [61], underscoring the direct relevance of our *in vitro *discoveries to human disease.

In addition to the proinvasive and MMP-2 activating properties associated with *Cxcl10 *in FLSs, this chemokine can also attract C-X-C chemokine receptor (CXCR)3-expressing inflammatory cells such as memory T cells [62] and mast cells [59] into the joint, further contributing to disease severity. Indeed, recent studies that either targeted *Cxcl10 *[63] or its receptor CXCR3 [64] significantly ameliorated arthritis in rodents.

*Cxcl10 *[65], *Vil2 *[66], and *Trim16 *[67] – three of the most significantly upregulated genes in DA – are known to be induced by estrogens (Figure 5). A complete analysis of all of the 66 differentially expressed genes revealed that nine of them (13.6%) were either regulated by estrogen (*Cxcl10*, *Vil2*, *Trim16*, *Gins3*, *Gadd45b*, and *Gmfg*) [68] or are involved in ER signaling (*Stip1*), ER ubiquitination (*Stub1*), or ER-mediated transcription (*Ncor1*). These observations suggested that abnormalities in the regulation of ER signaling and ER-mediated transcription could contribute to the invasive properties of DA FLSs. Indeed, estrogens have been shown to increase levels of active MMP-2 in various tissues and cell types [69-71], including breast cancers [72], and estrogen antagonists reversed that effect [71,73]. Estrogens also increase the production of active MMP-2 and the *in vitro *invasive properties of RA FLSs [74] (Figure 5). Although estrogens are typically thought of as having anti-inflammatory properties [75], our observations suggest an intrinsic dysregulation in ER signaling in DA FLSs. This dysregulation in ER is controlled by the *Cia5d *gene, and could contribute to increased FLS invasion and cartilage and bone erosive changes.

Five of the differentially expressed genes were located within the *Cia5d *interval, and this number was greater than expected by chance. Three of these were upregulated in DA.F344(Cia5d) FLS (*Ncor1*, *Trim41*, and *Gtlf3b*) and two were downregulated in DA.F344(Cia5d) (*Trpv2 *and *Trim16*), raising the possibility that a polymorphism/mutation in one of these genes could explain the arthritis and FLS invasive phenotypes attributed to *Cia5d*. Specifically, a polymorphism in a regulatory element or intron in one of these genes, or in another gene in the region, could influence transcription, thus explaining differences in levels of mRNA and disease. This has been the case in studies of two other autoimmune or inflammatory diseases in which microarray analysis led to the identification of the disease-causing polymorphism [76,77]. In the present study only *Ncor1*, a transcriptional repressor regulated by estrogens, appears to be an interesting candidate. *Trpv2 *is a cation channel ubiquitously expressed, and the other three genes (*Trim16*, *Trim41*, and *Gtlf3b*) have less clear functions. The *Cia5d *interval contains more than 100 genes, and not all were present in the Illumina microarray. It would be premature to exclude these genes at this point, and additional studies with recombinant subcongenic strains are under way.

## Conclusion

We have identified a novel invasion-associated gene expression signature and evidence suggesting a dysregulation in ER signaling in arthritis FLSs, which are regulated by the arthritis severity locus *Cia5d*. It is anticipated that the specific identification of the *Cia5d *gene, and the continued characterization of processes regulated by this gene, will generate new targets for therapeutic intervention aimed at reducing cartilage and bone destruction, and new prognostic markers for RA. The parallels between our findings in FLSs and observations from cancer studies suggest that the *Cia5d *gene might be important for cancer biology as well.

## Abbreviations

CXCR: C-X-C chemokine receptor; DMEM: Dulbecco's modified Eagle's medium; ER: estrogen receptor; FLS: fibroblast-like synoviocyte; GAPDH: glyceraldehyde-3-phosphate dehydrogenase; MMP: matrix metalloproteinase; MT1: membrane-type 1; PCR: polymerase chain reaction; RA: rheumatoid arthritis.

## Competing interests

The authors declare that they have no competing interests.

## Authors' contributions

All authors made substantial contributions to this study. TL generated the FLS cell lines and worked on the gene expression analyses. MB conducted the cellular and molecular biology experiments. WL worked on the gene expression statistical analysis. PSG designed the study and conducted the microarray gene expression analysis and pathway discovery, and wrote the manuscript. All authors read the manuscript critically, suggested modifications, and approved the final version.

## Ackowledgments

This study was funded by National Institutes of Health grants R01-AR46213, R01-AR052439 (NIAMS), and R01-AI54348 (NIAID) to Dr P Gulko. The authors want to thank Franak Batliwalla and Aarti Damle, members of the Feinstein Institute's microarray core facility, for their assistance.
